# New Tools for Data Harmonization and Their Potential Applications in Organ Transplantation

**DOI:** 10.1097/TP.0000000000005048

**Published:** 2024-05-17

**Authors:** Seyed Amir Tabatabaei Hosseini, Reza Kazemzadeh, Bethany Joy Foster, Emre Arpali, Caner Süsal

**Affiliations:** 1 Transplant Immunology Research Center of Excellence, Koç University Hospital, Istanbul, Turkey.; 2 Department of Pediatrics, McGill University, Montreal, QC, Canada.; 3 Department of Epidemiology, Biostatistics and Occupational Health, McGill University, Montreal, QC, Canada.; 4 Research Institute of the McGill University Health Centre, McGill University, Montreal, QC, Canada.

## Abstract

In organ transplantation, accurate analysis of clinical outcomes requires large, high-quality data sets. Not only are outcomes influenced by a multitude of factors such as donor, recipient, and transplant characteristics and posttransplant events but they may also change over time. Although large data sets already exist and are continually expanding in transplant registries and health institutions, these data are rarely combined for analysis because of a lack of harmonization. Promoted by the digitalization of the healthcare sector, effective data harmonization tools became available, with potential applications also for organ transplantation. We discuss herein the present problems in the harmonization of organ transplant data and offer solutions to enhance its accuracy through the use of emerging new tools. To overcome the problem of inadequate representation of transplantation-specific terms, ontologies and common data models particular to this field could be created and supported by a consortium of related stakeholders to ensure their broad acceptance. Adopting clear data-sharing policies can diminish administrative barriers that impede collaboration between organizations. *Secure multiparty computation* frameworks and the artificial intelligence (AI) approach *federated learning* can facilitate decentralized and harmonized analysis of data sets, without sharing sensitive data and compromising patient privacy. A *common image data model* built upon a standardized format would be beneficial to AI-based analysis of pathology images. Implementation of these promising new tools and measures, ideally with the involvement and support of transplant societies, is expected to produce improved integration and harmonization of transplant data and greater accuracy in clinical decision-making, enabling improved patient outcomes.

## Graphical abstract

**Figure:**
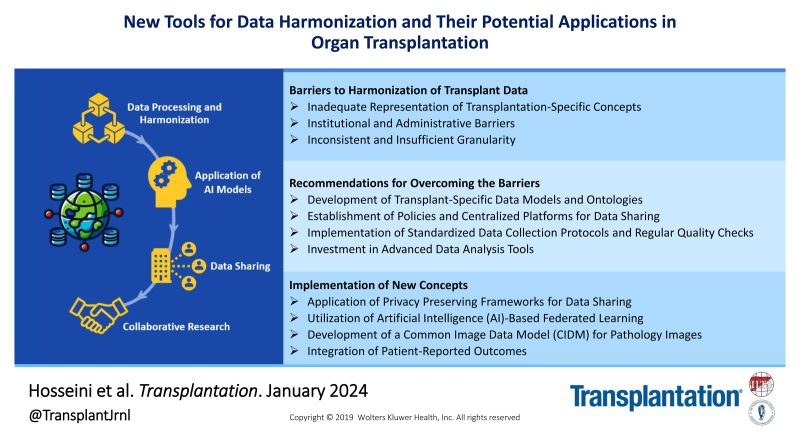

## INTRODUCTION

The digital transformation in healthcare has led to the accumulation of extensive data sets from various sources, including electronic health records (EHRs), patient registries, clinical trials, medical imaging, and proteomics and genomics analyses, often referred to collectively as *big data*.^1,2^ Such big data in healthcare encompass an array of structured and unstructured data sets that accumulate at high velocity and are characterized by their vast volume and wide variety.^3^ Effectively combining these data sets to conduct research holds tremendous potential to improve patient outcomes; however, they are typically stored separately and rarely shared, resulting in fragmented information.^4^ Moreover, data collection from health information systems often faces technical challenges, such as inconsistent data generation processes and inaccuracies including replications in the data sets.^5^ This disharmony in data structure causes delays in the comprehensive analysis of information and limits the ability to conduct multicohort analyses.^6^

Privacy concerns also pose a significant barrier, necessitating adherence to diverse legal, ethical, and regulatory frameworks to protect patient information.^7^ Breaching patient privacy can have severe consequences for both patients and healthcare providers. To overcome this obstacle, new solutions, organizations, and tools have emerged. Initiatives such as the Health Data Consortium work toward the ethical sharing of data, whereas technologies such as DataSHIELD offer methods for secure, privacy-preserving analysis across different data sets, promoting data consistency and integrity without compromising patient privacy.^8^

In organ transplantation, effective data harmonization is crucial because of the intricate interplay of factors and the need for large, high-quality data sets to accurately analyze continuously improving outcomes. In this overview, we describe the terminology and principles of data harmonization, present tools for data harmonization that emerged in other fields of healthcare and discuss the potential of these new tools for use in organ transplantation. By addressing the challenges of fragmented data, lack of standardization, privacy concerns, and technical constraints, these new technologies could revolutionize the analysis of organ transplant data.

## UNDERSTANDING DATA HARMONIZATION

Data harmonization is the process of merging data from various sources and standardizing it into a single format.

Prospective harmonization is the strategy of designing studies with the aim of attaining maximum harmonization from the outset.^9-11^ Conversely, retrospective harmonization is required when different data sources did not follow the same standards for all variables during the data collection process; harmonization must happen after data were collected. Registries use a prospectively harmonized language for data collection from the beginning, whereas meta-analysis of data from different registries often requires retrospective harmonization.^12^ In both cases, the degree of harmonization lies within a spectrum, and the ease of harmonization is influenced by established standards, compatibility, and practices that support the ability to repeat the process.

*Record linkage*, *data warehousing*, *data sharing*, and *health information exchange* are additional terms used to describe the complex process of data harmonization.^13-15^ Record linkage, in particular, refers to the amalgamation of data from diverse sources to establish a cohesive database. Typically, this involves the integration of data sets from the same individual, such as clinical and pathological data, through a unique identifier. Data warehousing refers to the storage of large amounts of data from different sources in a centralized location allowing easy access and analysis. Data sharing is the practice of making data accessible to other individuals and organizations, and health information exchange refers to sharing of health-related information among healthcare providers. The extent to which these efforts are similar in practice, scope, and relevance remains elusive and is still a point of discussion.

**FIGURE 1.**
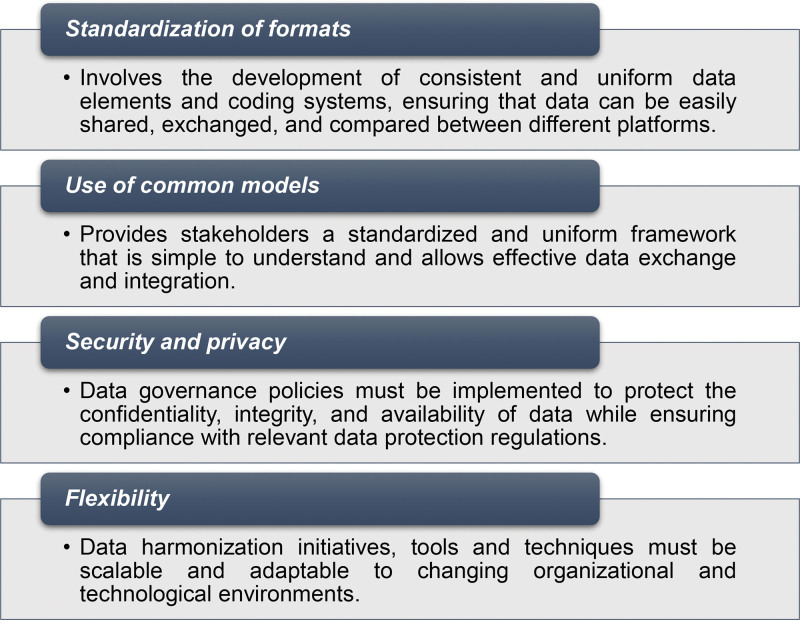
Key principles of data harmonization.

Data harmonization principles are guiding concepts that underlie the development and implementation of interventions aimed at harmonizing data across different platforms. By adhering to the principles shown in Figure 1, harmonization efforts can ensure that the data are accurate, consistent, and accessible across platforms.^16,17^ A standardized format for data fields, definitions, and coding systems is required for the consistency of collected data. One approach to achieve a high level of consistency is the use of a *common data model* (CDM). A CDM serves as a structured framework for defining data elements, their data collection protocols, and the variables that could be used to link data sets. CDM specifies how data should be structured, stored, and shared, ensuring uniformity across different data sets and sources. A CDM includes controlled vocabularies, which consist of standardized lists of phrases and codes, and it incorporates regular data quality checks to identify outliers and deviations from established standards. Developing a CDM typically involves a collaborative process, where experts in the field agree on the data elements, definitions, and coding systems that should be used. Data stored in separate databases using the same CDM will be uniformly collected and can be easily combined and analyzed. A *data governance policy*, which regulates data collection, sharing, storage, security, and privacy, is also vital. To ensure the long-term success of data harmonization initiatives, both CDMs as well as data governance policies must be flexible and able to evolve in line with changing requirements, technologies, and data sources.

## DATA HARMONIZATION INITIATIVES IN CLINICAL RESEARCH

Since the 1990s, there have been numerous efforts to standardize data collection and promote smoother harmonization. Table 1 presents a comprehensive compilation of organizations, projects, and tools for data integration and harmonization in clinical research, along with their initiation year, country of origin, aim, and application.

**TABLE 1.** Organizations, projects, and currently used tools for data harmonization in clinical research

Tool/project/organization		Year, place	Aim and application
ICD^18^	International Classification of Diseases	1990, WHO	To standardize classification and coding of diseases, injuries, and health conditions for medical record keeping, billing, and research purposes
HMORN^19^	Health Maintenance Organization Research Network	1994, the United States	To create a large-scale distributed network of health data
LOINC^20^	Logical Observation Identifiers, Names, and Codes	1994, the United States	To provide standardized terminology for laboratory and clinical observations to enable the electronic exchange of clinical data between healthcare entities
dm+d^21^	NHS Dictionary of Medicines and Devices	2000, the United Kingdom	National terminology in the United Kingdom for describing clinical concepts and enabling information exchange
SNOMED CT^22^	Systematized Nomenclature of Medicine Clinical Terms	2002, the United States	To provide a standardized clinical vocabulary for accurate and detailed documentation, communication, and analysis of clinical information
OMOP^23^	Observational Medical Outcomes Partnership	2008, the United States	To determine effective practices for retrospective observational research, create a CDM for transplant centers
AMT^24^	Australian Medicines Terminology	N/A, Australia	National terminology in Australia for describing clinical concepts and enabling information exchange
EHR4CR^25^	Electronic Health Records for Clinical Research	2011, Europe	To improve the design of patient-centric trials by accessing EHR systems, performing real-time queries using multiple clinical data warehouses, and receiving aggregated results
FHIR^26^	Fast Healthcare Interoperability Resources	2011, the United States	To standardize healthcare data exchange between different systems
EMIF^27^	European Medical Information Framework	2013, Europe	To enhance access to patient-level data from different health institutions across Europe and conduct multicohort studies on different diseases
PCORNet^28^		2014, the United States	To standardize data format and content for patient-centered outcomes research
OHDSI^29^	Observational Health Data Sciences and Informatics	2014, the United States	To develop methodologies to support large-scale observational studies with healthcare data, create a CDM to standardize healthcare databases and tools
i2b2-TranSMART^30^	Informatics for Integrating Biology and the Bedside	2017, the United States	To integrate patient data, perform cohort estimations, and determine study feasibility using anonymized EHR data
ICD-11^31^	International Classification of Diseases—11th version	2019, WHO	To address limitations of ICD-10, provide a more powerful health information system based on formal ontology, support future modifications, and compatibility with other classifications
N3C^32^	National COVID Cohort Collaborative	2020, the United States	To collect data from electronic health records for the study of COVID-19, harmonize and transform data from different CDMs into the OMOP common data model
DataHarmonizer^33^	N/A	2021, Canada	To address the data-sharing needs that arose during the COVID-19 pandemic. Enhance consistency, interoperability, and the capacity for repurposing contextual information throughout various data providers and sources

N/A, not applicable; WHO, World Health Organization.

### Initiatives to Standardize Terminology

One of the most important global initiatives in the harmonization of health data was the introduction of the International Classification of Diseases (ICD) by the World Health Organization in 1990, a standardized system for classifying and coding diseases, injuries, and other health conditions.^18^ The ICD is extensively used around the world for medical record keeping, billing, and research. Using standardized codes and categories, ICD-10 provides a common language that allows healthcare experts and researchers as well as other stakeholders to efficiently interact, exchange information, and analyze health data from multiple sources.^34^ Its latest version, ICD-11, captures disease characteristics more effectively than ICD-10, generates aggregated and more simplified representation of the data, supports multiple languages, and ensures data comparability with ICD-10.^20^ ICD-11 is a powerful health information system based on formal structure and designed for modern technology, allowing future modifications and compatibility with other classifications, and thus covering the main principles of data harmonization depicted in Figure 1.

Systematized Nomenclature of Medicine−Clinical Terms (SNOMED CT) and Logical Observation Identifiers Names and Codes (LOINC) in the United States, National Health Service Dictionary of Medicines and Devices in the United Kingdom, and Australian Medicines Terminology in Australia are further, widely used standards for harmonization of clinical terminology.^20-22,24^ SNOMED CT is a comprehensive clinical terminology system, designed for capturing detailed clinical concepts and relationships, making it suitable for clinical documentation in EHRs. It is highly granular and covers a wide array of clinical concepts, which is essential for detailed and precise clinical documentation. LOINC, on the other hand, is primarily focused on laboratory and clinical test results, providing a standardized way to represent observations and test data, thus making it suitable for harmonizing laboratory results and clinical measurements. Although ICD-11 is essential for billing, insurance claims, and public health statistics, SNOMED CT is often used for clinical coding, EHR systems, and clinical decision support, and LOINC is commonly used in laboratory information systems, clinical data exchange, and healthcare data compatibility. In practice, many healthcare organizations, especially in the US, use a combination of these systems to effectively capture different aspects of healthcare data. The choice of system or systems to use should align with the specific objectives and requirements of the data harmonization project.

### Projects to Integrate Healthcare Data From Different Sources

Initiated in 1994, the US Health Maintenance Organization Network^19^ is a large-scale decentralized network of interconnected healthcare databases for data sharing. Created in 2011, the European platform Electronic Healthcare Record for Clinical Research allows researchers to access EHR, run real-time queries against numerous warehouses of anonymized patient data, and receive aggregated results to help to design clinical trials.^25^ Initiated in the United States in 2017, the Informatics for Integrating Biology and the Bedside (i2b2-TranSMART) project, developed tools to merge patient data from different sources.^30^ One of its outcomes was a web application that could assess the feasibility of conducting clinical trials by identifying and estimating the size of eligible patient populations within the anonymized EHR data. However, because of the requirement of data centralization, this approach faced legal, ethical, and regulatory challenges.

### Standardizing Healthcare Data Through CDMs

Table 1 outlines also various initiatives that have introduced CDMs to standardize healthcare databases and facilitate the extraction, transformation, and loading of data.^23,28,29,32,35-37^ One pivotal initiative is the Observational Medical Outcomes Partnership (OMOP) which was established in 2008. OMOP originated as a collaborative effort between the public and private sectors, aimed at optimizing retrospective observational research by addressing the need for standardization in data formats, content, and methods. OMOP proposed and developed a CDM designed to enable effective implementation of retrospective observational studies by standardizing data representation across different healthcare databases.^38^

In 2020, amid the global COVID-19 pandemic, the National COVID Cohort Collaborative (N3C) in the US systematically collected data from EHRs for COVID-19 studies. Different medical institutions contributed data sets to N3C in four distinct CDMs. Leveraging the OMOP-CDM, the N3C data harmonization team devised a workflow to align definitions and transform the four CDMs into a unified format. This initiative illustrated how pre-existing tools such as OMOP could be repurposed and expanded to meet urgent public health needs, in this case, facilitating streamlined data analysis and research related to COVID-19.

OMOP is only one of the several CDMs developed to address specific needs within healthcare research. Although OMOP focuses on standardizing data to optimize retrospective observational studies, leveraging standardized data formats, content, and methods, National Patient-Centered Clinical Research Network (PCORNet), for instance, utilizes a distributed research network model to support patient-centered outcome research. PCORNet facilitates the aggregation of health data from various sources by engaging patients and stakeholders in the research process. Conversely, i2b2 aims to enhance translational research by integrating clinical information with biomedical data, employing a scalable informatics framework for querying and analyzing large-scale clinical and research data. The existence of multiple CDMs such as OMOP, PCORNet, and i2b2 reflects the diverse challenges and objectives in healthcare research, each offering a tailored approach to data standardization and harmonization for different research needs.

Furthermore, *mapping algorithms* have been developed that enable the translation and harmonization of data across different CDMs, leveraging the strength of each CDM. They translate data from one format to another and ensure that data sets from varied sources can be integrated and analyzed collectively. This process is instrumental in overcoming the challenges posed by the heterogeneity of healthcare data, paving the way for more comprehensive and nuanced analyses. A prominent example of mapping algorithms in action is seen in the *All of Us* Research Program of National Institutes of Health.^39^ The All of Us initiative has effectively utilized mapping algorithms to standardize data from multiple sources and aims to gather healthcare data from one million or more people living in the US to advance precision medicine.

**FIGURE 2.**
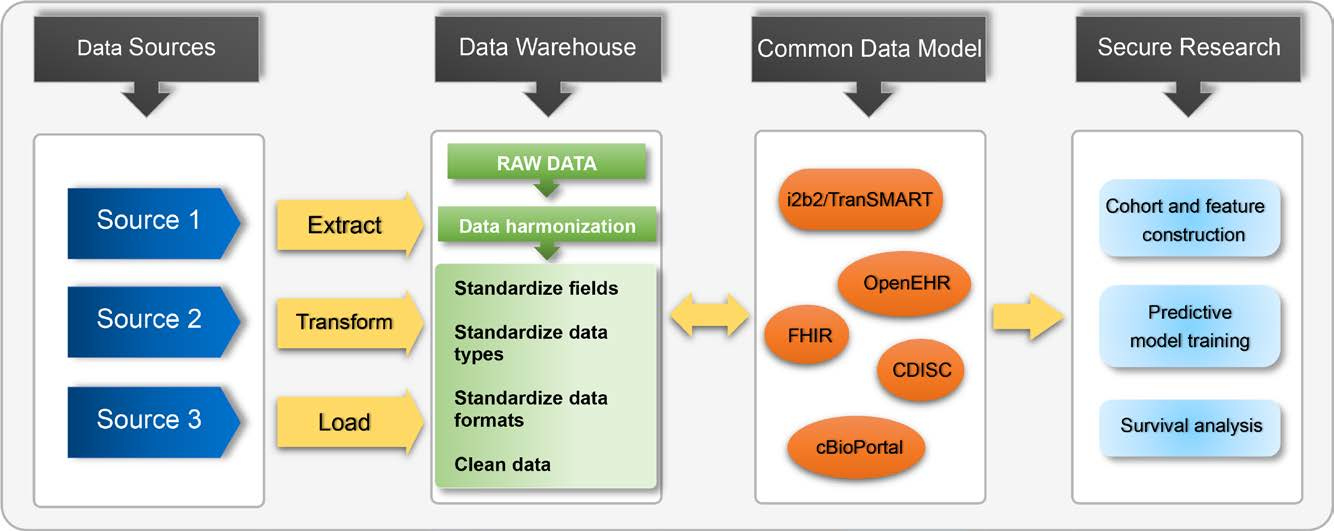
Visualization of a possible data preparation workflow for analysis of data from multiple sources. The procedure begins by obtaining data from different sources in various formats. Data are changed into a format that can be processed by a pipeline during the *Extraction* step. Concepts are automatically matched to their standard definitions using string similarity metrics as part of a semiautomated concept mapping process that takes place in the *Transformation* stage. An experienced clinical researcher who is familiar with the original concepts validates and approves the mappings that have been produced. The cohorts are finally loaded into the CDM databases during the *Loading* stage. Once the data are successfully loaded, they are ready for further analysis and exploration, including cohort and feature construction, predictive model training, and survival analysis. CDISC, Clinical Data Interchange Standards Consortium; CDM, common data model; FHIR, Fast Healthcare Interoperability Resources; i2b2-TranSMART, Informatics for Integrating Biology and the Bedside.

Figure 2 illustrates a possible workflow for processing and analyzing data from various sources using a CDM. The workflow includes stages such as data acquisition from different sources, extraction, and transformation of data with a process that involves both automated and manual steps for concept mapping, manual validation, and data loading into CDM databases. Once completed, the data is ready for in-depth analysis, providing valuable insights for clinical research.

### COVID-19’s Impact on Data Harmonization

The COVID-19 crisis served as a catalyst for data harmonization efforts. Global scientific communities collaboratively integrated diverse data sets, ensuring their compatibility and timely analysis, facilitating informed decision-making and effective public health responses. Besides the above-mentioned N3C initiative that harmonized and transformed EHRs and used a CDM for streamlined COVID-19 research,^32^ the DataHarmonizer,^33^ a tool freely available on GitHub, was developed to address data-sharing needs that arose during the COVID-19 pandemic. DataHarmonizer offers data standardization, enforces validation rules, aggregates diverse data sources, facilitates in-depth analysis, and has potential to enhance data harmonization in other fields of healthcare research, including organ transplantation.

## DATA HARMONIZATION INITIATIVES IN ORGAN TRANSPLANTATION

### Transplant Registries

The Collaborative Transplant Study, initiated in 1982 by Opelz et al,^40^ stands as a cornerstone in collection of harmonized transplant data. This multinational scientific venture amassed data from >800 000 transplants across 5 organ types, including their combinations and spanning >400 centers worldwide.^40^ The study paved the way for national and international registries such as the US Scientific Registry of Transplant Recipients and the Australia and New Zealand Dialysis and Transplant Registry. These registries play a vital role, continuously replenishing the transplant community with crucial information on patient demographics, procedures, outcomes, and risk factors. However, the linkage of existing national and international registries to each other and other data repositories require further collaboration and harmonization efforts. Inconsistent data collection methods can affect data quality and completeness. Limited data accessibility and inadequate analytical tools may hinder research, whereas initiatives such as online reporting tools offer promising steps toward improved access. Additionally, inconsistencies in data upkeep, both across and within individual registries over time, can present challenges for robust analysis. Incomplete follow-up information, outdated entries, and variations in data collection practices in different periods can further complicate research efforts. The recent formation of the Transplantation Society’s Global Data Harmonization Committee in the late 2020 signifies a pivotal step toward addressing these challenges. The committee aims to provide guidance to the Transplantation Society Executive and Council on matters related to transplant data availability and harmonize data collection and analysis efforts across different regions and registries. Key initiatives include development of common data standards, standardization of data elements and terminology, facilitation of interregistry data linkage, promotion of data-sharing policies, and collaboration with relevant organizations and stakeholders.

### Standards, Guidelines, and Ontology-based Approaches for Harmonization of Organ Transplant Data

The Clinical Data Interchange Standards Consortium (CDISC) plays a crucial role in establishing standards and guidelines for data harmonization in clinical research. Specifically, CDISC has developed a Therapeutic Area User Guide-Kidney Transplantation which includes disease-specific metadata and guidance on implementing CDISC standards for various purposes such as data collection, analysis, and reporting.^41^

Ontologies, serving as formal representations of knowledge with a common vocabulary and shared understanding of concepts, facilitate seamless linkage and interoperability across data sources. They contribute to the precise collection and exchange of data on specific topics. An example of such an ontology is the Ontology for Biomedical Investigations (OBI), which provides a framework for representing and harmonizing data across multiple biomedical domains, including organ transplantation.^42^ Several projects and resources, such as ImmPort, The National Center for Biomedical Ontology Annotator, The Encyclopedia of DNA Elements, and Human Immunology Project Consortium, are utilizing OBI for annotating experimental data, representing experimental protocols, and enhancing data integration and harmonization.

However, it is crucial to clarify how 1 OBI and other similar ontologies can effectively be adopted. Researchers and transplant centers can integrate OBI into their data management processes by aligning their data structures and terminologies with OBI’s standardized concepts. This alignment ensures that data generated or utilized by researchers adheres to the structure and terminology defined by OBI. In practical terms, incorporating OBI may involve the mapping of existing data to OBI concepts or adjusting data collection protocols to align with OBI standards. This adoption process ensures that the data generated are not only consistent and structured but also interoperable with other data sets using the same ontology. A tangible example of successful adoption is evident in the ImmPort platform managed by the National Institutes of Health. ImmPort serves as an archive for immunology research data and effectively harmonizes organ transplantation data. This is achieved by providing detailed annotations using standardized concepts and ontologies such as OBI.^43^

## BARRIERS TO HARMONIZATION OF ORGAN TRANSPLANT DATA

Inadequate representation of transplantation-specific concepts, institutional and administrative barriers, and inconsistent granularity represents severe hurdles that hinder effective data harmonization in organ transplantation.

### Inadequate Representation of Transplantation-specific Concepts

The major problem of data harmonization in organ transplantation is that the currently available tools for this purpose lack the ability to accurately represent concepts specific to the field. Transplantation involves complex allocation-related calculations including priority, active/inactive status, and exception points for disadvantaged patients. Changing pathological criteria over time and complex donor characteristics, such as infectious risk donor, kidney donor risk index, kidney donor profile index, and expanded criteria donor, and recipient parameters, such as HLA mismatch, model for end-stage liver disease (MELD), and life years from transplant, and so on, further add to the complexity of transplant data.

An example of inadequate representation of transplantation-specific concepts becomes evident in the analysis of mortality during the waiting period for a liver transplant using scoring systems and indices such as MELD. MELD score must be calculated uniformly across different transplant centers; however, existing general vocabularies often lack adequate representation of transplant-specific concepts, and various scoring systems and indices such as MELD are applied differently in different databases, resulting in noninterpretable scales. This diversity in calculation methods poses challenges for combining databases seamlessly for comprehensive evaluation of patient outcomes, organ quality, or risk factors. Transplant centers adhere to different protocols with visits at varying time points after transplant, frequently record free-text observations in different languages, and rarely utilize structured data formats, such as ICD codes.^44,45^

### Institutional and Administrative Barriers

Effective collaboration in organ transplant data collection is often hindered by conflicting institutional policies, inadequate data governance, and varying data privacy rules, especially across multiple countries with distinct regulatory frameworks. Existing systems for data capture, such as EHR systems like Epic or Cerner, frequently lack compatibility capacity, which complicates harmonization efforts. Inadequate data governance and stewardship practices further intensify the challenges of harmonizing organ transplant data. Although there is acknowledgment of the necessity for international standards and guidelines governing organ transplant data collection, storage, and sharing, there are limited incentives to alter current data capture approaches.

The challenges and rewards of data harmonization vary considerably between national and international efforts. Nationally, the harmonization of organ transplant data grapples with specific challenges, including the variability in health system operations over time, differences in reimbursement policies for transplant procedures, and the diversity in treatment and allocation policies across regions. These factors introduce a level of complexity because of their dynamic nature; for instance, health system operations may evolve in response to technological advancements or changes in healthcare legislation, whereas reimbursement policies could change according to economic conditions or shifts in healthcare priorities. Similarly, treatment and allocation policies are subject to updates based on new medical evidence or changes in ethical guidelines, further complicating the landscape for data harmonization. Moreover, the changes in established classifications and the diversity in requirements across different departments within the healthcare system can lead to significant disparities in how data are collected, managed, and interpreted. This variability not only makes it challenging to maintain consistency in data collection but also impedes the ability to achieve uniformity in data analysis across different transplant centers. For example, a change in the classification of a certain type of organ donation might be adopted at different times or in varying manners by different centers, leading to discrepancies in data that can undermine the reliability of national transplantation statistics. The repercussions of these challenges include redundant data entry, the maintenance of parallel data management systems, and overlapping data analysis procedures, which not only increase the workload for healthcare professionals but also inflate the costs associated with data management.

At the international level, the nuances in each country’s approach to health data security and patient privacy add another layer of complexity to harmonization efforts. This complexity is further compounded by varying organ allocation policies between countries, which can significantly affect the comparability of data and the ability to conduct meaningful cross-country analyses.

The benefits of data harmonization at the national level are substantial. The adoption of harmonized data formats across transplant centers enables the generation of more meaningful statistics and fosters deeper insights into transplantation trends and outcomes within the country. By ensuring that data are collected and managed in a consistent manner, healthcare professionals and policymakers are better equipped to identify areas for improvement, allocate resources more effectively, and implement policies that enhance the efficiency and efficacy of organ transplantation services. At the international level, the rewards of harmonization are even more significant, offering a broader perspective on organ transplant practices and outcomes and enhancing global understanding and innovation in this critical medical field. Through the availability of more comparable, consistent, and coherent data, international harmonization efforts facilitate global research collaborations, contribute to the advancement of medical science in organ transplantation, and ultimately lead to improved global health outcomes.

Overall, national and international data harmonization efforts, albeit challenging, are crucial for fostering research in organ transplantation and improving global health outcomes.

### Inconsistent and Insufficient Granularity

The complexity of achieving uniform, consistent, and comparable granularity across multiple data sources is a further notable barrier to harmonization of transplant data. Data may be gathered with varied degrees of specificity, for example, by documenting merely the occurrence or absence of a single symptom rather than its intensity or frequency. For example, if a research study aims to analyze the impact on kidney transplant outcomes of donor-recipient HLA matching at epitope level, inconsistencies in data granularity can lead to situations where the detailed two-field HLA typing information required for this analysis on 11 different HLA loci is available only from a few centers, whereas the information from the majority of centers will be limited to only 3 loci at 1-field antigen level. This variability in data granularity would hinder the ability to accurately assess the role of HLA epitope matching in kidney transplant success and may delay the introduction of this possibly more effective matching process in organ allocation.

The barriers to progress in data harmonization have significant consequences for transplant recipients, transplant centers, and the healthcare system. A detailed assessment of how these barriers impact various entities involved in organ transplantation is shown in Table 2, and a detailed breakdown of the challenges encountered in harmonizing organ transplantation data is illustrated in **Table S1** (**SDC**, http://links.lww.com/TP/D56). Organ-specific barriers to data harmonization and their potential solutions are highlighted in **Table S2** (**SDC**, http://links.lww.com/TP/D56).

**TABLE 2.** Impact of data harmonization barriers on organ transplantation entities

Affected entity	Description
Patient outcomes	Inadequate data representation and granularity limitations hinder assessment of outcomes, particularly in small patient groups such as children. Suboptimal organ allocation, imprecise treatment strategies, and the inability to harness precision medicine can result in suboptimal patient care and reduced transplant success rates
Resource management	The lack of comprehensive data impedes efficient resource management within transplant centers. This hinders effective allocation of resources, leading to potential inefficiencies and increased healthcare costs
Quality of care	Inconsistent data quality and granularity can lead to incorrect decision-making in patient care and hinder quality assurance efforts, impacting patient safety and treatment outcomes
Global collaboration	Barriers in data sharing and harmonization hinder global collaboration and the identification of best practices. This lack of collaboration can result in missed opportunities to improve the quality of care on a global scale and to reduce disparities
Equity in access to transplantation	The inability to address transplant-related disparities because of data limitations can affect equity in access to transplantation
Policy and system efficiency	Absence of evidence-based data hampers development of effective policies and regulations for the healthcare system. This, in turn, affects the efficiency of healthcare delivery and resource allocation

## RECOMMENDATIONS FOR ADDRESSING THE LIMITATIONS OF DATA HARMONIZATION IN ORGAN TRANSPLANTATION

A comprehensive set of recommendations for addressing the above-mentioned challenges to data harmonization in organ transplantation is presented in Table 3.

**TABLE 3.** Recommendations for overcoming the shortcomings of data harmonization in organ transplantation

Shortcoming	Recommendations
Inadequate representation of unique concepts	Develop and implement organ-specific data models
	Create and maintain a transplantation-specific ontology, regularly updated to reflect clinical practice changes and new discoveries
	Establish a consortium with representatives from multiple institutions and stakeholders to develop and maintain the data models and ontologies, ensuring widespread adoption and consistent application across institutions and data sources
	Utilize a comprehensive common guideline for timing, frequency, and nature of data collection
Institutional and administrative barriers to collaboration	Establish clear and standardized data-sharing policies and procedures for transplant institutions, including guidelines for data security, privacy, and confidentiality
	Develop and implement a standard data use agreement for all institutions, facilitating data sharing while safeguarding patient privacy and confidentiality
	Invest in training and education on collaboration, data management, sharing, and analysis in transplantation for researchers, clinicians, and administrators
	Establish a centralized data repository or sharing platform exclusively for transplant institutions, ensuring accessibility and user-friendliness for stakeholders
	Implement standardized coding frameworks or mapping algorithms to ensure accurate and consistent data analysis, enabling evidence-based decision-making and promoting collaboration in global health research and policy
Missing granularity	Implement standardized data collection and reporting protocols to consistently capture comprehensive data across all institutions and sources
	Conduct regular audits and quality checks to identify and address missing or incomplete transplantation data elements in collaboration with institutions and data sources
	Invest in the development and widespread adoption of advanced data analysis and visualization tools capable of handling complex transplantation data, ensuring accessibility for all stakeholders

Developing organ-specific CDMs and ontologies that evolve with changes in clinical practice is crucial to rectify the problem of inadequate representation of unique concepts. Fostering collaboration among multiple institutions and organizations as well as stakeholders is essential for the widespread adoption and consistent application of such CDMs and ontologies. An international guideline for data collection at follow-up visits at predefined, standardized time points would be helpful. To establish a stronger foundation for collaborative efforts in organ transplantation and circumvent institutional and administrative barriers, stakeholders including transplant societies, healthcare institutions, governmental bodies, data management organizations, and researchers should address issues related to data governance and stewardship through unambiguous policies, standardized data use agreements, and training initiatives. The establishment of a centralized data repository would be ideal. This proactive approach will ensure the successful harmonization of data across diverse healthcare landscapes, recognizing the multifaceted nature of challenges in data harmonization initiatives. Granularity issues can be tackled by implementing standardized data collection protocols including routine quality checks and investing in advanced data analysis and visualization tools.

### Stakeholders That Can Drive Progress in Organ Transplantation Data Harmonization

In the realm of organ transplantation, the journey toward effective data harmonization is a collaborative effort involving a diverse array of stakeholders. At the forefront of this collective endeavor are transplant centers, serving as the frontline hubs for data collection and management. Transplant centers can actively contribute to the progress of data harmonization by adopting standardized practices, sharing data, and engaging in collaborative initiatives. Professional associations and transplant societies with their expertise and resources can offer guidelines on best practices and educational materials that encourage the standardization of data. Regulatory bodies that oversee healthcare practices can play a crucial role by establishing guidelines that promote harmonization and ensuring adherence of centers to consistent data collection and reporting standards. Government agencies, researchers, clinicians, technology providers, patients, advocacy groups, educational institutions, and data experts all play pivotal roles in this ecosystem, collectively fostering an environment where data harmonization can thrive. Table 4 provides a concise overview of the various stakeholders and their possible specific contributions to the progress of data harmonization in organ transplantation.

**TABLE 4.** Stakeholders that can facilitate progress in data harmonization

Stakeholder	Description
Transplant centers	Transplant centers as frontline hubs for data collection and management can actively support harmonization through standardized practices, data sharing, and collaboration
Professional associations and transplant societies	Can provide expertise and resources and offer platforms, guidelines, best practices, and educational materials to encourage data standardization among their members
Regulatory bodies	Can establish guidelines and regulations to promote harmonization, ensuring consistent data collection and reporting standards across healthcare practices
Government agencies	Can promote data sharing and harmonization at a national level; incentivize centers to participate in initiatives and fund research in data harmonization
Researchers and clinicians	Can actively participate in harmonization initiatives, advocate for best practices, and collaborate on research projects to obtain high-quality, harmonized data
Technology providers	Can design and implement solutions that support transplant centers in standardization of data collection, storage, and sharing
Educational institutions	Can offer training programs and courses on data management, harmonization, and informatics, equipping healthcare professionals with skills to support harmonization
Data scientists and analysts	Can contribute expertise to develop advanced data analysis and visualization tools, ensuring effective utilization of harmonized data for research and decision-making

## FUTURE PERSPECTIVES

As advancements in technology continue to shape the healthcare landscape, there is growing anticipation regarding the potential utilization of several methods to improve data sharing and harmonization in organ transplantation. Table 5 outlines these methods, suggesting their future applicability in enhancing the organ transplant data harmonization process.

**TABLE 5.** Concepts with potential to improve data harmonization in organ transplantation

Key concepts	Description
Privacy-preserving frameworks	Tools and frameworks, such as DataSHIELD, Differential Privacy, TEEs, and Secure Data Enclaves, can facilitate collaborative analysis of diverse data sets while protecting the privacy and confidentiality of individuals
AI-based federated learning	Federated learning, an AI technique, enables decentralized model training while preserving data privacy. This approach fosters collaborative model improvement, aiding in optimization of organ allocation strategies and enhancement of patient outcomes
CIDMs	By providing protocols for image acquisition and quality assurance, CIDMs can reduce variations and enhance the reliability of AI-based image analysis in organ transplantation research
Integration of patient-reported outcomes	Harmonizing and incorporating patient-reported outcomes like quality of life into the data sets can provide a fuller picture of transplantation impacts

AI, artificial intelligence; CIDMs, common image data models; TEEs, trusted execution environments.

### Implementation of Privacy-preserving Frameworks

Data privacy-preserving frameworks allow researchers to perform analyses on distributed data sets collected and stored at different locations, such as hospitals, research institutions, and registries, without physically moving or centralizing the data.^46^ The distributed data analysis ensures that data remain under the control of the data custodians, maintaining the privacy and security of individual-level information. These frameworks employ privacy-preserving methods, such as *secure multiparty computation* and cryptographic protocols, to perform analyses on encrypted or aggregated data. Researchers share hereby, instead of sensitive individual-level data, only the necessary summary statistics or aggregate results. Creating such frameworks for organ transplantation can facilitate the harmonization of diverse data sets from multiple centers and the statistical analyses performed locally on each site, without compromising data privacy and creating additional issues that arise post data sharing, such as unauthorized access, data misuse, ownership disputes, over time impaired data quality, changing regulations, and loss of control, community trust, and reputation.^47^

DataSHIELD is the most prominent tool that adopts this concept of taking the analysis to the data, not the data to the analysis, ensuring data owners and officers retain control over the data.^8^ It allows co-analysis of individual-level data from multiple studies or sources without the need for physical transfer of the actual data. *Differential privacy*, *trusted execution environments*, and *secure data enclaves* are additional tools, frameworks, and environments that serve a similar purpose.^48-50^ Use of these tools offer enhanced privacy and security measures and could improve data sharing and harmonization efforts in organ transplantation.

### Implementation of Artificial Intelligence-based Federated Learning

**FIGURE 3.**
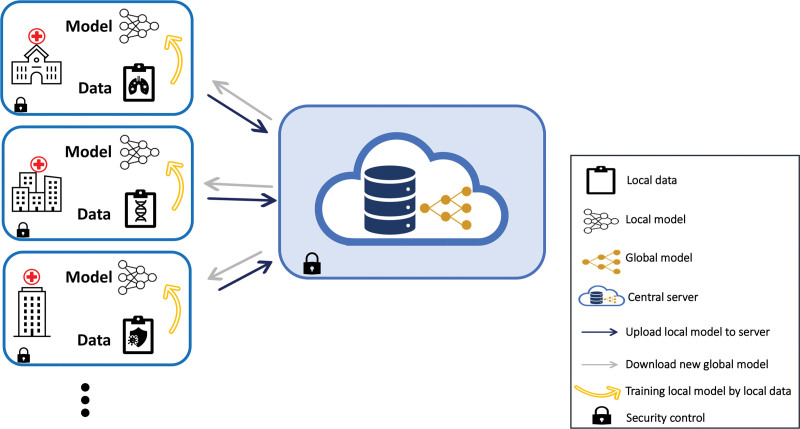
Workflow of the federated learning system. Initially, a central server distributes the initial model to the participating sources. Each source then trains the model, making small improvements to the model using its own local data. After training, instead of sending the data back to the central server, the source sends only the model updates (gradients) that capture the changes made during the training process. The server combines these updates from multiple sources to update the global model. This process iterates several times, with the model improving gradually through collaboration.

Artificial intelligence (AI) methods are expected to play a significant role in the future of data harmonization in organ transplantation. *Federated learning* as an AI approach enables training of machine learning models on decentralized data sources. Figure 3 illustrates the working principles of a federated learning system. Instead of transferring raw patient data across institutions, the models are trained at each center on local data and only the model updates are sent to the central server. The central server combines these updates from multiple sources to optimize the global model, allowing harmonized analysis. This process iterates several times, with the global model improving gradually through collaboration. By aggregating insights from multiple transplant centers, this privacy-preserving technique can be utilized to build predictive models, for example, to optimize allocation strategies and improve patient outcomes.

### Development of a Common Image Data Model

Although the analysis of pathology images plays a critical role in monitoring graft health and guiding treatment decisions, sharing and analysis of images across multiple institutions present significant challenges because of differences in staining methods, file formats, and image quality. AI tools are increasingly used in image analysis and AI algorithms trained on images from 1 center may not work well on images from other centers, introducing biases and artifacts that affect the reliability and consistency of AI-based analyses.

**FIGURE 4.**
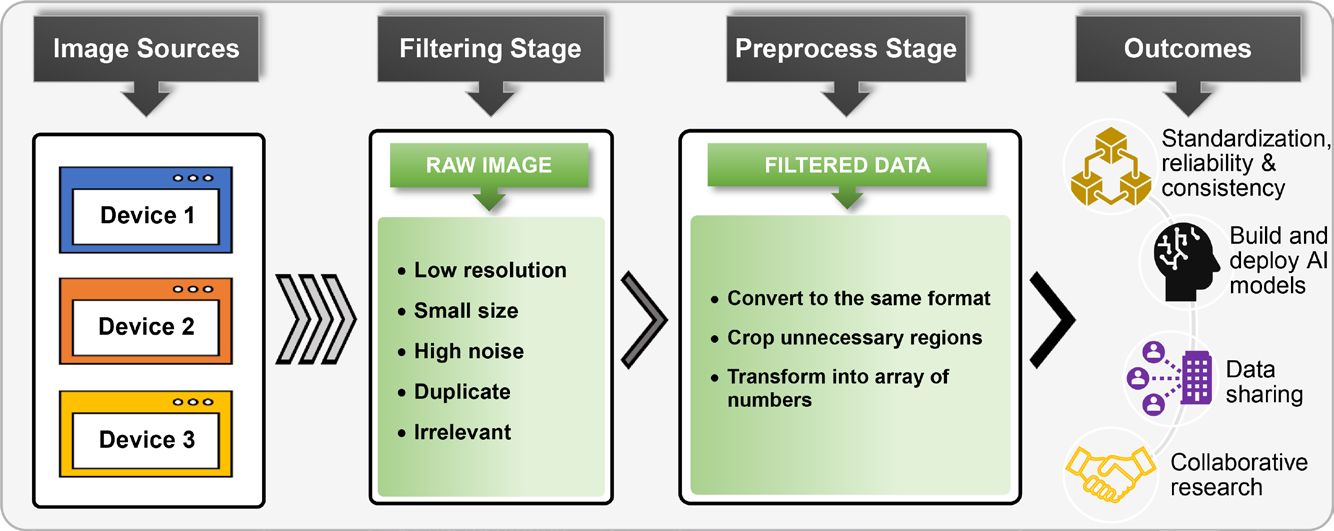
Visualization of a possible CIDM workflow for preparing pathological and radiological images collected from multiple sources. The process initiates by obtaining data from different sources or devices in various formats. When collecting images, it is common to encounter low-resolution, improperly sized, noisy, duplicate, or irrelevant images. The *Filtering* step aims to eliminate these undesirable images, culminating in the construction of a valuable image data set. Filtered images are automatically converted to the standardized format, unnecessary regions within each image are cropped, and the regions of interest are transformed into arrays of numbers as part of the *Preprocess* stage. An experienced clinical researcher reviews the images and validates the data set that has been produced. Once the data set’s reliability is confirmed, it becomes ready for sharing or further analysis, including AI-based exploration and evaluation. AI, artificial intelligence; CIDM, common image data model.

Creation of a *common image data model* (CIDM) that defines consistent image formats, metadata standards, and quality control measures can help to address this challenge by standardizing the representation, storage, and exchange of pathological images across transplant centers. Standardization would enable seamless sharing and collaboration, ensuring that AI tools trained on a common data set are more reliable and accurate in their analysis across multiple centers. Furthermore, the development of standardized protocols and guidelines for image acquisition, preprocessing, and quality assurance can help to minimize the impact of variations in image quality. Implementing quality control measures and regularly calibrating imaging systems can enhance the consistency and comparability of pathology images for AI-based analyses. Figure 4 depicts a potential workflow outlining the various stages involved in a CIDM for preparation of pathology data, facilitating both data sharing and advanced analysis.

### Addressing the Gaps in Patient-reported Outcomes

The integration of long-term, patient-reported outcomes such as quality of life into transplant data by the existing large registries remains insufficient. This represents a significant gap because such outcomes are indispensable for evaluating the success of transplantation from the patient’s perspective to convincingly demonstrate the long-term benefits of transplantation. Such evidence may reduce potential recipients’ hesitancy toward transplantation and highlight the socioeconomic benefits of enabling recipients to return to work.

## CONCLUSIONS

Improved data harmonization across organ transplantation data sets holds great promise to improve knowledge. The main challenges that currently obstruct successful data harmonization in organ transplantation are inadequate representation of specific concepts, insufficient data granularity, and institutional and administrative obstacles to cooperation. Addressing these challenges through transplant-specific data models and ontologies, standardized protocols for data collection and sharing, and the adoption of new tools for data analysis and visualization can significantly improve data quality and analysis. Furthermore, implementing privacy-preserving frameworks such as DataSHIELD and secure data enclaves can enable collaborative analysis of disparate data sets protecting individual’s privacy and confidentiality. Incorporating AI-based techniques such as federated learning into harmonization initiatives can facilitate the development of more reliable predictive models and risk stratification algorithms. Similarly, a CIDM could facilitate the sharing and analysis of pathological images between separate institutions. Incorporation of patient-reported outcomes, such as quality of life, into the data harmonization efforts would help to demonstrate the full spectrum of transplantation benefits. In conclusion, deploying these strategies and the use of novel harmonization tools can completely transform the field of organ transplantation, leading to a dramatic improvement of data quality and analysis and, in the consequence, success rate of transplantations.

## ACKNOWLEDGMENTS

The authors gratefully acknowledge the use of the services and facilities of the Koç University Research Center for Translational Medicine (KUTTAM), funded by the Presidency of Turkey, Head of Strategy and Budget.

## Supplementary Material


