# Supplementary material for: New Tools for Data Harmonization and Their Potential Applications in Organ Transplantation
Source: Transplantation. 2024 May 17;108(12):2306–17. doi: 10.1097/TP.0000000000005048 (PMC11581435; doi:10.1097/TP.0000000000005048)
Supplement: Supplementary file 1 [file tpa-108-2306-s001.pdf]

Table S1. Challenges in harmonization of organ transplantation data.

Areas Hindered by Data Limitations	Description
**Outcome Analysis in Small Patient Groups**	Lack of power hinders the ability to study outcomes in smaller groups of patients. Harmonization across datasets is necessary to have adequate power to assess outcomes in smaller subgroups such as children.
**Analysis of the Cause of Graft Failure and Mortality**	Missing data in existing datasets and inconsistent data collection across datasets can hinder the analysis of the cause of graft failure and mortality.
**Outcome Analysis Across Countries**	Inconsistent outcome reports can hinder comparison of the outcomes across countries.
**Optimization of Organ Allocation**	Non-harmonized data can affect the evaluation of allocation systems, resulting in suboptimal organ distribution.
**Development of Global Strategies**	Variability in data privacy regulations and coding systems across countries restricts data exchange preventing opportunities to discover and implement global strategies.
**Precision Medicine**	Varying levels of granularity in different data sources may hinder accurate assessment of complex influential factors such as donor-recipient HLA matching, impeding development of personalized treatment strategies.
**Predictive Models**	Without comprehensive data, it is challenging to create accurate predictive models that can assist in decision-making to improve outcomes.
**Quality Assurance**	Inconsistent data quality across institutions and data sources can undermine quality assurance efforts, leading to errors in clinical decision-making.

Table S2. Organ-specific barriers to data harmonization.

Organ	Barriers	Potential Solutions
**Heart**	Heterogeneity of data sources and collection methods (electronic health records, data from echocardiograms, cardiac catheterizations, etc.)	Standardization of data collection protocols and guidelines across different data sources
	Variability in data formats and coding standards (ICD-10, LOINC)	Consistent data coding standards and terminology across different data sources
	Complexity of cardiac anatomy and physiology	Advanced data modeling techniques and machine learning algorithms to extract meaningful insights from complex cardiac data.
**Liver**	Lack of standardized definitions for liver disease severity and outcomes	Establishment of consensus definitions for disease severity and outcomes through international multidisciplinary collaboration
	Variability in liver biopsy interpretation	Standardized biopsy scoring systems and training programs for pathologists
	Limited availability of genetic and molecular data	Collection and integration of genetic and molecular data into liver transplant registries
**Kidney**	Difficulty in attributing outcomes to specific characteristics of kidney transplants	Methods for robustly attributing outcomes to kidney transplants, considering donor and recipient characteristics and transplant procedures
	Lack of standardized measures of kidney function	Implementation of standardized measures of kidney function such as estimated glomerular filtration rate (eGFR) across different data sources
	Limited availability of data on long-term transplant outcomes	Enhanced follow-up practices and data collection to capture long-term outcomes, including graft and patient survival and quality of life
**Lung**	Heterogeneity of lung disease phenotypes	Standardized classifications and characterizations of lung disease phenotypes
	Variability in lung function testing methods	Harmonized lung-function testing protocols and guidelines
	Lack of standardized measures of lung transplant outcomes	Consensus definitions and standardized measures of survival, lung function, and quality of life
**Intestine**	Limited availability of data	Increased collection and sharing of data through registries and collaborative research initiatives
	Lack of standardized definitions for transplant success	Consensus definitions for graft survival, enteral nutrition independence, and quality of life
	Variability in transplant protocols	Standardized protocols and guidelines to improve consistency
